# 3‐Methylglutaconyl‐CoA hydratase deficiency: When ascertainment bias confounds a biochemical diagnosis

**DOI:** 10.1002/jmd2.12332

**Published:** 2022-09-14

**Authors:** Ashley Hertzog, Arthavan Selvanathan, Dinusha Pandithan, Won‐Tae Kim, Maina P. Kava, Avihu Boneh, David Coman, Adviye Ayper Tolun, Kaustuv Bhattacharya

**Affiliations:** ^1^ NSW Biochemical Genetics Service, Western Sydney Genetics Program The Children's Hospital at Westmead Westmead New South Wales Australia; ^2^ Disciplines of Genetic Medicine and Child and Adolescent Health The University of Sydney Sydney New South Wales Australia; ^3^ Genetic Metabolic Disorders Service Sydney Children's Hospital Network Sydney New South Wales Australia; ^4^ Queensland Lifespan Metabolic Medicine Service Queensland Children's Hospital Brisbane Queensland Australia; ^5^ Department of Metabolic Medicine The Royal Children's Hospital Parkville Victoria Australia; ^6^ NSW Newborn Screening Programme The Children's Hospital at Westmead Westmead New South Wales Australia; ^7^ Metabolic Unit, Department of Rheumatology and Metabolic Medicine Perth Children's Hospital Perth Western Australia Australia; ^8^ School of Paediatrics and Child Health University of Western Australia Perth Western Australia Australia; ^9^ Department of Paediatrics University of Melbourne Parkville Victoria Australia; ^10^ School of Medicine University of Queensland Brisbane Queensland Australia

**Keywords:** 3‐Methylglutaconyl CoA hydratase deficiency, C5OH, inborn error of metabolism, leucine catabolism, MGA1

## Abstract

3‐Methylglutaconyl‐CoA hydratase deficiency (MGA1) is a defect in leucine catabolism, which causes the accumulation of urinary 3‐methylglutaconate, with or without 3‐hydroxyisovalerate and 3‐methylglutarate. It is an ultra‐rare condition, with <30 cases published in the literature. It is unclear whether the clinical features seen in reported patients are caused by the biochemical abnormalities, or whether they simply represent an ascertainment bias in patients that come to clinical attention. We reviewed the collective Australian experience of patients with confirmed MGA1, four of whom were diagnosed when asymptomatic through newborn screening (NBS). When our cohort is considered alongside the broader literature, there is no clear evidence of a specific childhood‐onset clinical phenotype associated with this disorder. Some patients have non‐specific clinical features (such as autism spectrum disorder [ASD]); however, there are also other family members with ASD in the absence of MGA1, suggesting a multifactorial aetiology. Importantly, all four patients diagnosed through NBS (including three with over 18 years of clinical follow‐up) remain asymptomatic in the absence of treatment. Based on the available literature, we suggest that MGA1 represents a biochemical phenotype, with an absence of a childhood clinical phenotype. The burdens of sustained treatment (particularly with intensive dietary leucine restriction) in asymptomatic individuals may be of little benefit, and likely to result in poor compliance. Longer‐term follow‐up of patients detected via NBS (or biochemical screening of large cohorts of asymptomatic adult individuals) will be required to conclusively prove or disprove the association with adult‐onset leukoencephalopathy.

## 1 INTRODUCTION

3‐Methylglutaconyl‐CoA hydratase (EC4.2.1.18), encoded by the *AUH* gene, catalyses one of the key steps in leucine catabolism. Bi‐allelic pathogenic variants in the *AUH* gene impair the function of this enzyme, causing elevations of urinary 3‐methylglutaconate (3‐MGCA), with or without 3‐hydroxyisovalerate (3‐HIVA) and 3‐methylglutarate (3‐MG).^1^ The clinical consequences of these urinary abnormalities have not been fully established in the limited literature (<30 patients).^2^, ^3^, ^4^, ^5^ Historically, 3‐methylglutaconyl‐CoA hydratase deficiency (OMIM no. 250950; also known as 3‐methylglutaconic aciduria Type 1 [MGA1]) was thought to be a benign biochemical abnormality, given many patients had normal development in childhood.^1^, ^6^ However, more recent reports suggest a late‐onset phenotype of leukoencephalopathy.^7^, ^8^, ^9^

Given this increasing evidence of a late‐onset phenotype, development of an evidence‐based treatment strategy merits consideration. Variable combinations of dietary restriction, carnitine supplementation and high‐calorie feed during catabolic stress have been trialled.^5^, ^6^, ^9^ These treatment strategies were already being utilised prior to the recognition of a late‐onset phenotype. Whilst this resulted in correction of the biochemical abnormalities in some cases, it is difficult to evaluate the clinical benefits of treatment compared with those who were untreated.

We present seven patients with a confirmed diagnosis of MGA1, including the long‐term follow‐up of two previously reported patients.^1^ We describe their clinical course, biochemical profile, and genotype. This case series adds to the body of literature suggesting that this disorder is asymptomatic in childhood.

## 2 METHODS

Patients with MGA1 were collated by contacting all Australian metabolic centres. Epidemiological data, clinical information, diagnostic biochemistry and molecular results were compiled by retrospective chart review (Table 1). Urine organic acids (performed by gas chromatography mass spectrometry) and plasma acylcarnitines (performed by liquid chromatography tandem mass spectrometry) were analysed using established local protocols. Patients had either enzymatic or molecular confirmation of MGA1. Two patients with 3‐MCGA who are siblings of confirmed patients with MGA1 are also included.

**TABLE 1:** Clinical, biochemical, and molecular features of Australian patient cohort

		Patient 1	Patient 2	Patient 3	Patient 4	Patient 5	Patient 6	Patient 7
Epidemiology	Current age	16 months	18 years	5 years	7 years	24 years	21 years	19 years
	Age at diagnosis	2 weeks	1 month	2 weeks	2 years	3 years	2 weeks	2 weeks
	Gender	Male	Female	Male	Female	Female	Male	Female
	Consanguineous (Y/N)	N	N	N	N	Y	Y	Y
Symptoms at diagnosis	Language delay (Y/N)	N	N	Y	Y	Y	N	N
	Motor delay (Y/N)	N	N	N	N	Y	N	N
	Failure to thrive (Y/N)	N	N	N	N	N	N	N
	Optic atrophy (Y/N)	N	N	N	N	N	N	N
	Ataxia (Y/N)	N	N	N	N	Y	N	N
	Other clinical features at diagnosis	Asymptomatic	Asymptomatic	Hypotonia	Intermittent diarrhoea, expressive language delay	Tremors; also had an acute encephalopathy after a febrile illness.	Asymptomatic	Asymptomatic
Presenting biochemical features	Detected via newborn screening (Y/N)	Y	Y	N	N	N	Y	Y
	Plasma C5OH	0.63 μmol/l (RR: <0.1)	—	0.15 μmol/l (RR: 0.1–0.15)	—	—	0.3 μmol/l (RR: <0.1)	—
	Urine 3‐methylglutaconate	Gross elevation	Gross elevation	Gross elevation	Moderate elevation	Moderate elevation	Gross elevation	Gross elevation
	Urine 3‐hydroxyisovalerate	BDL	BDL	Trace elevation	Slight elevation	Moderate elevation	BDL	BDL
	Urine 3‐methylglutarate	BDL	Slight elevation	—	—	—	BDL	BDL
	Enzyme activity	Not performed	Reduced	Not performed	Not performed	Reduced	Reduced1	Not performed
Radiology	Age at MRI	Not performed	Not performed	1 month	Not performed	3 years; 13 years	Not performed	Not performed
	Leukoencephalopathy (Y/N)	—	—	N	—	Y (at 13 years)	—	—
	Other findings	—	—	Small lactate peak	—	Acute demyelination at 3 years	—	—
Molecular diagnosis	Variants in AUH	c.260del|p.Arg87Glnfs*8, c.80del|p.Ser27Metfs*8	c.620 T > G|p.Leu207Arg, c.676C > T|p.Arg226Cys	c.719C > T|p.Ala240Val, c.668G > A|p.Arg223Gln	c.719C > T|p.Ala240Val, c.668G > A|p.Arg223Gln^a^	Homozygous c.262G > C|p.Gly88Arg	Homozygous c.80del|p.Ser27Metfs*8	Homozygous c.80del|p.Ser27Metfs*8^a^
	Exon (NM_001698.3)	Exon 1/Exon 1	Exon 6/Exon 7	Exon 7/Exon 7	Exon 7/Exon 7	Exon 1	Exon 1	Exon 1
Treatment	Leucine restriction (Y/N)	N	N	N	N	N	N	N
	L‐carnitine supplementation (Y/N)	N	N	N	N	Y	N	N
Progress and outcomes	Language delay (Y/N)	N	N	Y	Y	Y	N	N
	Autism spectrum disorder (Y/N)	N	N	Y	Y	N	N	N
	Motor delay (Y/N)	N	N	N	N	Y	N	N
	Failure to thrive (Y/N)	N	N	N	N	N	N	N
	Optic atrophy (Y/N)	Not assessed	N	N	N	Not assessed	Not assessed	Not assessed
	Ataxia (Y/N)	N	N	N	N	Y	N	N
	Other clinical features	None	None	Neutropenia developed at 1 month of age; resolved by 10 months of age. Atopy	Obstructive sleep apnoea (awaiting adenotonsillectomy). Left eye esotropia. Intermittent fevers	Recurrent otitis media, precocious puberty, pigmented teeth, bicuspid aortic valve. Childhood neglect	None	None

Abbreviation: BDL, below limit of detection; MRI, magnetic resonance imaging; RR, reference range.

^a^ Genotype assumed.

### 2.1 Patient 1

Patient 1 was identified on newborn screening (NBS) with an elevated 3‐hydroxyisovalerylcarnitine of 1.7 μmol/l (reference range [RR]: <1). Plasma acylcarnitine analysis confirmed this elevation (0.63 μmol/l; RR: 0–0.1). Urine organic acid analyses at 2 and 3 weeks of age showed a persistent gross elevation in 3‐MGCA, with elevations of 3‐HIVA and 3‐MG present in the repeat sample.

Massively parallel sequencing (MPS) analysis of genes associated with 3‐methylglutaconic acidurias detected bi‐allelic pathogenic variants in Exon 1 (c.80del |p.Ser27Metfs*8 and c.260del |p.Arg87Glnfs*8) of the *AUH* gene.

He is now 15 months of age and, at this stage, there are no concerns regarding his growth or development and does not receive any active treatment.

### 2.2 Patient 2

Patient 2 was identified on NBS with an increased C5OH. Subsequently, urine organic acid analysis demonstrated a gross elevation in 3‐MGCA, a slightly elevated 3‐MG and trace amounts of glutarate.

An estimate of 3‐methylglutaconyl‐CoA hydratase activity was performed on fibroblast analysis utilising a coupled enzyme assay. This indicated a defect in leucine degradation distal to 3‐methylcrotonyl‐CoA, with a considerably skewed 3‐hydroxybutyrate to Coenzyme A ester ratio (0.1, around 3%–4% of controls).

Sequencing of all 10 exons of the *AUH* gene confirmed Patient 2 was compound heterozygous for variants in Exon 6 (c.620 T > G|p.Leu207Arg) and Exon 7 (c.676C > T|p.Arg226Cys).

Throughout childhood, she had normal growth and development. She is currently 18 years of age and remains asymptomatic without any active treatment.

### 2.3 Patient 3 (Proband)

Patient 3 was investigated for neonatal hypotonia and neutropenia at 1 month of age. Urine organic acid analysis demonstrated a gross elevation in 3‐MGCA with a slight elevation in 3‐HIVA. Plasma acylcarnitine analysis showed a C5OH level at the upper limit of normal (0.15 μmol/l; RR: 0–0.15). Brain magnetic resonance imaging (MRI) did not identify any white matter changes; however, a small lactate peak was observed on spectroscopy.

MPS analysis of a panel of genes associated with 3‐methylglutaconic acidurias identified bi‐allelic pathogenic variants (c.668G > A | p.Arg223Gln and c.719C > T | p.Ala240Val) in Exon 7 of the *AUH* gene.

Patient 3's hypotonia and neutropenia resolved by 1 year of age. He is now 4 years old and has been diagnosed with autism spectrum disorder (ASD; Level II) and atopy (eczema, allergic rhinitis and urticaria). He receives no active treatment for his biochemical diagnosis.

### 2.4 Patient 4 (Sister of Patient 3)

Patient 4 (older sister of Patient 3) was subsequently investigated at 4 years of age. Urine organic acid analysis showed a gross elevation in 3‐MGCA with a slight elevation in 3‐HIVA.

She was noted to have fatigue and gut dysmotility. She later developed intermittent exotropia and recurrent unexplained fevers. She is currently 6 years of age and has also recently been diagnosed with ASD (Level II). She receives no active treatment for her biochemical diagnosis.

### 2.5 Patient 5

Patient 5 was a child of Indigenous origin, born of consanguineous parents. She presented with encephalopathy at 3 years of age, in the context of a febrile illness. She had pre‐existing mild global developmental delay. Ataxia and tremors were noted after recovery. An MRI scan of the brain demonstrated symmetrical changes consistent with acute demyelination. Urine organic acid analysis identified a moderate elevation in urinary 3‐MGCA and 3‐HIVA. Enzyme studies in cultured skin fibroblasts (similar to those mentioned above) demonstrated a skewed 3‐hydroxybutyrate to Coenzyme A ester ratio (0.1; RR: 0.9–11.3). Molecular sequencing identified a likely pathogenic homozygous variant (c.262G > C) in *AUH*.

She is now 24 years old with a mild intellectual disability and persistent ataxia. She had one further episode of drowsiness following a fall at age 4, with elevated lactate, but has otherwise not had any further episodes of encephalopathy. A repeat MRI scan showed leukoencephalopathy with relative sparing of subcortical U‐fibres, the periventricular white matter and the corpus callosum.

Her other medical conditions include a bicuspid aortic valve with ascending aortic dilatation, renal calculi, juvenile idiopathic arthritis, central precocious puberty and immunoglobulin G3 deficiency. It is unclear whether any of these issues are related to MGA1; however, she is at risk of other monogenic conditions given her consanguineous heritage. She takes carnitine supplementation (100 mg/kg/day).

### 2.6 Patient 6 (Proband)

Patient 6 was identified on NBS with an elevated C5OH. Plasma acylcarnitine analysis confirmed this elevation in C5OH (0.3 μmol/l; RR <0.1), and there was a gross increase in 3‐MGCA on urine organic acid analysis. 3‐HIVA and 3‐MG were not increased initially, though there were slight elevations in these metabolites at 2 months of age. The C5OH level normalised after 3 months and remained normal at 2 years of age. As previously reported,^1^ the diagnosis of MGA1 was confirmed on enzymatic analysis of skin fibroblasts.

Patient 6 had no issues in the newborn period and continued to have normal development throughout childhood. He is now 21 years of age, completed mainstream high school and is enrolled in a university degree. He continues to play multiple sports, attends the gym regularly and has elected to take high‐protein sports supplements. He receives no active treatment.

### 2.7 Patient 7 (Sister of Patient 6)

Patient 7 (the younger sister of Patient 6) had a raised C5OH on NBS. Urine organic acid analysis showed gross increase in 3‐MGCA, with 3‐HIVA and 3‐MG not increased. Given the diagnosis in her older brother, further confirmatory testing via enzymatic or molecular analysis was not pursued.

Patient 7 has similarly remained asymptomatic since birth; at Age 19 she has also enrolled in a university degree with no concerns regarding cognition and plays multiple sports. She receives no active treatment.

## 3 DISCUSSION

The 3‐methylglutaconic acidurias are a clinically and biochemically heterogeneous group of disorders. Primary elevation of 3‐MGCA occurs in defects of leucine catabolism, most notably 3‐methylglutaconyl‐CoA hydratase deficiency (MGA1), but also from deficiency of the more distal enzyme, 3‐hydroxy‐3‐methylgutaryl CoA Lyase (HMGCL).^10^ As a result, increased 3‐MGCA excretion in response to leucine loading is characteristically seen in MGA1.^11^, ^12^ 3‐Methylglutaconic aciduria can also arise from defects that cause mitochondrial dysfunction, in which the 3‐MGCA is a secondary finding.

Early reports of MGA1 were of patients that had clinical symptoms and signs. Greter et al.^13^ published two siblings with a slowly progressive encephalopathy (dementia, spastic paraparesis, dyskinesias and optic atrophy) that began from 6 months of age. Urinary markers were further increased on loading with leucine and a diagnosis of MGA1 was postulated. However, subsequently, Narisawa et al.^14^ demonstrated normal 3‐MGCH enzyme activity in these patients. It is similarly difficult to confirm diagnoses of MGA1 in other patients with 3‐methylglutaconic aciduria from this time period, given the lack of confirmatory enzymatic and molecular genetic analysis.

We reviewed the literature regarding all patients with molecularly or enzymatically confirmed MGA1 (summarised in Table S1). Nardecchia et al.^2^ recently summarised clinical, biochemical, and molecular data from 20 such patients. Additionally, we have identified three patients from the literature,^3^, ^4^, ^5^ as well as our own five previously unreported cases. Of these 28 patients, 25 were diagnosed in childhood and three in adulthood. In total, 9 of 25 children (36%) in this cohort did not have any intellectual or developmental deficit, along with the three adults who did not present in childhood. A total of 14 patients (50%) had MRIs, and 12 of these (86%) had variable abnormalities, including nine children. The degree of increases of the key metabolites (3‐MGCA, 3‐HIVA and 3‐MG) was variable; however, the vast majority of reported patients excreted increased concentrations of all three metabolites. 3‐MG appeared to be the metabolite which was most variable, ranging from below the limit of detection in some patients to grossly increased in others. This may reflect variation in patient metabolism or laboratory analysis.

In the context of this clinical, biochemical and neuroradiological variability, further elucidation of the pathogenesis underlying the disorder is necessary. There is speculation that 3‐HIVA is the toxic compound associated with leukoencephalopathy seen in patients with MGA1. Engelke et al.^8^ proposed this mechanism, citing evidence of raised CSF/plasma ratios of 3‐HIVA in an adult patient with MGA1 and progressive leukoencephalopathy, but also in other conditions affecting leucine degradation where neurological abnormalities occur (such as biotinidase deficiency).^15^ However, there are reports of patients with normal MRIs, who are still excreting 3‐HIVA.^12^, ^16^ Additionally, another disorder with raised 3‐HIVA, 3‐methylcrotonyl‐CoA carboxylase (3‐MCC) deficiency, does not result in neurological or developmental abnormalities in at least 57% of patients.^17^ 3‐HIVA is also excreted in isovaleric acidaemia and HMGCL deficiency, neither of which have the same reported phenotype as MGA1. This suggests in patients with MGA1 who have MRI lesions, other mechanisms must be involved, including factors such as rate of renal clearance of (or blood–brain barrier permeability to) 3‐HIVA, or altered activity of other enzymes involved in leucine catabolism.

In terms of treatment, within this previously published cohort, 14 patients were leucine‐restricted or protein‐restricted, 9 were prescribed L‐carnitine supplementation and 3 received increased calories when unwell. Three patients specifically received no treatment and it was unclear if an additional four were treated (or not). Additionally, it is difficult to ascertain the efficacy of treatment as there is limited longitudinal follow‐up. The burdens of sustained treatment (particularly with intensive dietary leucine restriction) in asymptomatic individuals may be of little benefit and result in poor compliance.

More than half of our Australian cohort were identified on NBS with elevated C5OH levels. Importantly, three patients (Patients 2, 6 and 7, the latter two of whom have been previously reported^1^, ^9^) were diagnosed on NBS, received no treatment, and are now asymptomatic adults. Patients 3 and 4 both have ASD, and they also have a sibling without 3‐methylglutaconic aciduria who has ASD. This highlights that ascertainment bias may also be leading to a perception of causality in historical cases, especially with mild phenotypes (slight speech delay or learning difficulties) where there could be alternative explanations. Only one patient in our Australian cohort (Patient 5) receives treatment (dietary leucine restriction and carnitine supplementation).

MGA1 has generally been ascertained as an incidental finding following a positive C50H on NBS (a biomarker screened to primarily identify other conditions such as HMGCL deficiency). NBS has now identified MGA1 in individuals who are asymptomatic to adult life without treatment. Even for those patients that have been identified in the investigation of developmental delay, there is no definitive evidence of effective treatment. This does not fulfil the Wilson and Jungner^18^ diagnostic criteria for screening; therefore, it is difficult to mount a case to specifically perform NBS for this disorder.

Long‐term clinical follow‐up for those who have been diagnosed incidentally by NBS could be considered to monitor for the reported late manifestations; whether or not these can be prevented (or treated) remains uncertain. Given the rarity of this disorder, our descriptive study has limitations due to the small number of patients. Larger patient cohorts would also be beneficial in understanding disease pathophysiology and the need for treatment.

## 4 CONCLUSION

There appear to be two distinct phenotypes associated with MGA1: a seemingly benign, biochemical disorder detected in the childhood period and a late‐onset leukoencephalopathy. It is unclear whether the former phenotype progresses to the latter, or they occur in separate groups of patients. In our case series, the majority of patients (including those who are now adults) remain asymptomatic despite the lack of therapeutic intervention. This is also true for all four patients identified on NBS; however, follow‐up has not extended into late adulthood. Given this uncertainty, we would advocate for semi‐regular monitoring of patients identified in childhood. Further studies are needed to clarify whether the biochemical findings lead to late‐onset leukoencephalopathy, and to conclusively determine whether commencing treatment at the time of such neurological involvement is beneficial.

## AUTHOR CONTRIBUTIONS

**Ashley Hertzog:** planning of project, submission of ethics approval, performing laboratory investigations, performing literature review, drafting and editing article, submission process and correspondence with journal. **Arthavan Selvanathan:** literature review, clinical management of patients and drafting and editing article. **Dinusha Pandithan:** clinical management of patients and drafting and editing article. **Won‐Tae Kim:** performing laboratory investigations and drafting and editing article. **Maina P. Kava:** clinical management of patients and drafting and editing article. **Avihu Boneh:** clinical management of patients and editing article. **David Coman:** clinical management of patients and editing article. **Adviye Ayper Tolun:** drafting and editing article. **Kaustuv Bhattacharya:** performing literature review, clinical management of patients and drafting and editing article.

## CONFLICT OF INTEREST

This article has not been submitted for review elsewhere and has been approved by all other authors for submission.

## ETHICS STATEMENT AND PATIENT CONSENT

All procedures followed were in accordance with the ethical standards of the responsible committee on human experimentation (institutional and national) and with the Declaration of Helsinki of 1975, as revised in 2000. This project has received approval from the local human research ethics committee (CCR2021/19 and 2021/ETH01044), and signed consent (or waivers of consent) was obtained.

## ANIMAL RIGHTS

This article does not contain any studies with animal subjects performed by any of the authors.

## Supporting information


**TABLE S1** Supporting Information

## Data Availability

The data from this case series is confidential patient information and, as such, not publicly available. However, the authors are contactable regarding case specifics.
